# Blocking Cross-Species Secondary Binding When Performing Double Immunostaining With Mouse and Rat Primary Antibodies

**DOI:** 10.3389/fnins.2021.579859

**Published:** 2021-05-25

**Authors:** Shanping Mao, Guoxiang Xiong, Brian N. Johnson, Noam A. Cohen, Akiva S. Cohen

**Affiliations:** ^1^Department of Neurology, Renmin Hospital, Wuhan University, Wuhan, China; ^2^Department of Anesthesiology and Critical Care Medicine, Children’s Hospital of Philadelphia, Philadelphia, PA, United States; ^3^Philadelphia Veterans Affairs Medical Center, Philadelphia, PA, United States; ^4^Departments of Otorhinolaryngology—Head and Neck Surgery, Perelman School of Medicine, University of Pennslyvania, Philadelphia, PA, United States; ^5^Department of Anesthesiology and Critical Care Medicine, Perelman School of Medicine, University of Pennsylvania, Philadelphia, PA, United States

**Keywords:** cross immunoreactivity, immunohistochemistry, fluorescent staining, rodent, guinea pig

## Abstract

Immunostaining is a powerful technique and widely used to identify molecules in tissues and cells, although critical steps are necessary to block cross-reaction. Here we focused on an overlooked cross immunoreactivity issue where a secondary antibody (secondary) cross-reacts with a primary antibody (primary) from a different species. We first confirmed the previously reported cross-species binding of goat anti-mouse secondary to rat primary. This was accomplished by staining with a rat primary against glial fibrillary acidic protein (GFAP) and visualizing with goat (or donkey) anti-mouse secondary. We then further revealed the converse cross-species binding by staining with a mouse primary against neuronal nuclear protein (NeuN) and visualizing with anti-rat secondaries. We speculate that mouse and rat primaries share antigenicity, enabling either secondary to recognize either primary. To block this cross-species binding in double staining experiments, we compared three protocols using mouse anti-NeuN and rat anti-GFAP, two primaries whose antigens have non-overlapping distributions in brain tissues. Simultaneous staining resulted in cross-species astrocytic staining (anti-mouse secondary to rat anti-GFAP primary) but no cross-species neuronal staining (anti-rat secondary to mouse anti-NeuN primary). Cross-species astrocytic staining was missing after sequential same-species staining with mouse anti-NeuN primary, followed by rat anti-GFAP. However, cross-species astrocytic staining could not be diminished after sequential same-species staining with rat anti-GFAP primary, followed by mouse anti-NeuN. We thus hypothesize that a competition exists between anti-mouse and anti-rat secondaries in their binding to both primaries. Single staining for NeuN or GFAP visualized with dual secondaries at different dilution ratio supported this hypothesis.

## Introduction

Immunostaining is a powerful tool for identifying molecules in tissues (immunohistochemistry) or cells (immunocytochemistry) and has been used in many studies. In neuroscience research, for instance, immunostaining is particularly useful when searching for disease biomarkers (Gentleman et al., 1993; Sherriff et al., 1994). Except for direct (and perhaps less sensitive) staining using a fluorescent dye- or chromagen-conjugated primary antibody (primary), a secondary antibody (secondary) is necessary in most immunostaining protocols (Berzofsky et al., 2003; Buchwalow and Böcker, 2010). This methodology is rooted in the notion that the primary is serving as an antigen to be recognized by the secondary and the secondary is thought to be specific to the primaries raised from the target animal species (Berzofsky et al., 2003; Buchwalow and Böcker, 2010). For example, a goat or donkey anti-mouse secondary should recognize only primaries produced from the mouse and ideally, and should not recognize primaries from any other species, such as rat.

When selecting and using primary antibodies it is well understood that the specificity of the primary antibodies for their intended antigen must be rigorously confirmed (Lorincz and Nusser, 2008; Saper, 2009), due to the possibility of primary antibody binding to off-target molecules in cells or tissues (Xiong et al., 2014; Mao et al., 2016). Non-specific binding by a secondary antibody can often be adequately blocked by incubating the target tissue in normal serum of the animal species from which the secondary antibody was derived, e.g., normal goat serum must be used when visualization is performed with a goat anti-mouse or anti-rat secondary. Unfortunately, less attention is often paid to the specificity of the secondary antibody for the intended primary antibody. This lack of species specificity can be especially problematic, as the secondary antibody may bind in a non-random, specific way, but to primary antibodies from a species other than the target species. When double staining is performed for two different target molecules simultaneously, two different primaries raised in different host animal species are usually used, with the hope that doing so will prevent the same secondary from binding to both primaries. Surprisingly, some secondaries have been shown to exhibit cross-species binding to foreign-species primaries. For instance, a goat anti-*mouse* secondary might recognize *rat* primaries, and a goat anti-rabbit secondary may cross-react to primaries raised from guinea pig (Erickson et al., 1993).

In the present study, we sought to develop a solution to the problem of the cross-species binding of a secondary to the off-target primary. We performed immunofluorescent staining using a rat monoclonal antibody to glial fibrillary acidic protein (GFAP), and a mouse monoclonal against neuronal nuclear protein (NeuN, or A60 as in Mao et al., 2016). This approach was implemented because the respective antigens are exclusively present in astrocytes or neurons, respectively, and do not have overlapping patterns of expression. As such, it was easy to ascertain when there had been unintended secondary-primary cross-species binding. We began with single staining experiments designed to determine the following: (1) if the cross species binding between goat anti-*mouse* secondary and *rat* anti-GFAP primary could be reproduced using our established protocol (Xiong et al., 2012; Mao et al., 2016); (2) if donkey anti-mouse secondary, like goat anti-mouse secondary, would also cross react with rat anti-GFAP primary; and (3) if there was a cross-species binding between anti-*rat* secondary and *mouse* anti-NeuN primary. We then compared three different double staining protocols, and developed a method to block the cross-species secondary binding when staining with both mouse anti-NeuN and rat anti-GFAP primaries.

As an alternative way to solve the problem of secondary antibody cross-species binding, it has been proposed that using a secondary pre-adsorbed with normal IgG from the animal of the interfering “cross species” can block the cross-species binding (Erickson et al., 1993). That is, cross-species binding of anti-mouse secondary to rat primary might be blocked if the anti-mouse secondary was pre-adsorbed on a column in which normal rat IgG was immobilized. To test this proposal (Erickson et al., 1993), cross-species pre-adsorbed goat anti-mouse and goat anti-rat secondaries were also tested in single and double staining experiments.

## Materials and Methods

In the present study, we used 8-week-old male C57Bl/6 mice from Jackson Laboratory (Bar Harbor, ME). The procedures and protocols for all animal studies were approved by Institutional Animal Care and Use Committees of Wuhan University, Children’s Hospital of Philadelphia, and University of Pennsylvania, in accordance with international guidelines on the ethical use of animals (National Research Council, 1996). To ensure the reproducibility of our findings, each experiment was conducted at a minimum on slices from at least three animals.

Mice were deeply anesthetized with 0.5 ml of 5% chloral hydrate and perfused with 15 ml of 0.1 M phosphate buffered saline (PBS) followed by 50 ml of freshly made 4% paraformaldehyde (PFA) in 0.1 M phosphate buffer at room temperature (RT). Brains were removed and post-fixed in the same PFA solution for 90 min at RT. Frontal slices were cut in 0.01 M PBS (pH 7.4) at 50 μm in thickness with a Leica VT 1000s vibratome (Leica Microsystems Inc., Buffalo Grove, IL). To minimize the number of animals used, six series of slices from each brain were collected in PBS for different staining with an interval of 300 μm between two adjacent slices within an identical series (Xiong et al., 2012).

To assess cross-species secondary binding, we began by performing single immunofluorescent staining using a free-floating slices protocol (Xiong et al., 2012; Mao et al., 2016). All primary and secondary antibodies used in the present study are detailed in Table 1. Slices were permeabilized with 0.3% triton X-100 and non-specific staining was blocked with a mixture of 1% bovine serum albumin (BSA) and 5% normal goat or donkey serum (NGS or NDS), depending on the species from which the secondary was raised. After incubation with rat primary against GFAP (IgG form; Unpurified hybridoma supernatant; 1:2; A generous gift from Dr. Judith B. Grinspan, Children’s Hospital of Philadelphia) for 60 min at RT and then overnight at 4°C, visualization was done using Alexa Fluor dye-conjugated goat (or donkey) anti-mouse IgG secondary [1:200; Jackson Immuno Research (West Grove, PA) or Invitrogen (Carlsbad, CA)] for 75 min at RT, together with Hoechst (Invitrogen) to counter-stain all nuclei in the brain slices. When mouse primary against NeuN (IgG form; 1:500; Millipore-Sigma, Burlington, MA) was used, cross-species visualization was done with Alexa Fluor dye-conjugated goat (or donkey) anti-rat IgG secondary (1:200; Jackson Immuno Research or Invitrogen; Table 1). In addition to immunostaining for cross species secondary binding, same-species secondaries (goat or donkey anti-rat secondary for GFAP and goat or donkey anti-mouse secondary for NeuN) were also used in order to show the normal species-specific staining pattern. To determine if the anti-mouse and anti-rat secondaries have competitive binding abilities to mouse or rat primaries, separate slices were incubated with rat anti-GFAP or mouse anti-NeuN primary, and then visualized with a mixture of goat anti-rat and goat anti-mouse secondaries at comparative dilution ratios of 1:1 (both 1:200) or 1:5 (1:100 for anti-rat and 1:500 anti-mouse). A thorough wash with PBS was performed after primary incubation and secondary visualization, in order to ensure no unbound free antibodies remained in slices.

**TABLE 1:** List of antibodies used in the present study.

Mouse primary	Monoclonal mouse anti-NeuN (Clone A60), Purified IgG, Millipore, MAB377
Rat primary	Monoclonal rat anti-GFAP, Hybridoma supernatant, Dr. Judith B. Grinspan
Rabbit primary	Monoclonal rabbit anti-NeuN, Purified IgG, Abcam, ab177487
Anti-mouse secondary	*AF488-GxM, Jackson Immuno Research, 115-545-003 AF594-GxM, Jackson Immuno Research, 115-585-003 AF488-GxM, Invitrogen, A-11001
	AF488-DxM, Jackson Immuno Research, 715-545-150
Anti-rat secondary	AF594-GxRt, Jackson Immuno Research, 112-585-003 AF488-GxRt, Jackson Immuno Research, 112-545-003 AF594-GxRt, Invitrogen, A-11007
	AF594-DxRt, Jackson Immuno Research, 712-585-150
Anti-rabbit secondary	AF488-GxRb, Jackson Immuno Research, 111-545-003
Highly adsorbed secondary	AF488-GxM (with Rt IgG), Jackson Immuno Research, 115-545-166
	AF594-GxRt, (with mouse IgG), Jackson Immuno Research, 112-585-167

*AF: Alexa Fluor dyes conjugated to secondary antibodies. DxM (Rt), Donkey anti-mouse, or -rat secondary; GxM (Rb or Rt), Goat anti-mouse, -rabbit, or -rat secondary.

As part of our effort to block cross-species secondary binding when staining the same brain slice with both mouse anti-NeuN and rat anti-GFAP primaries, we tested three protocols using simultaneous (Xiong et al., 2012; Mao et al., 2016) or two sequential applications of both primaries. For simultaneous staining, the slices were incubated with a mixture of rat (anti-GFAP) and mouse (anti-NeuN) primaries, and then visualized with a mixture of anti-rat and anti-mouse secondaries. If the binding ability were similar among the four possible primary-secondary pairs, then either primary might be bound by both anti-mouse and anti-rat secondaries to produce same- and cross-species staining during simultaneous visualization. In the first sequential staining protocol, slices were incubated with mouse anti-NeuN primary and visualized with anti-mouse secondary, then incubated with rat anti-GFAP primary and visualized with anti-rat secondary (see Table 2 for the step-by-step protocol). The second sequential staining reversed the order of the first protocol, and completed the rat anti-GFAP primary staining and anti-rat secondary visualization, followed by mouse anti-NeuN primary staining and anti-mouse secondary visualization. In either sequential protocol, the first secondary was for same-species visualization of the first primary. Since no unbound free antibodies (from the first round) remained prior to the second round, concentration of the later secondary would be much higher during the nominal “same-species” visualization. As a negative control for cross-species interaction, simultaneous staining was performed using a *rabbit* primary against NeuN [1:1,000; Abcam, Cambridge, MA; Mao et al., 2016) and the rat anti-GFAP primary, as no cross-species interaction has been noticed between rabbit and rat.

**TABLE 2:** Step by step protocol for sequential staining to avoid cross immunoreactivity using both mouse and rat primary antibodies.

**Day 1**	**Day 2**	**Day 3**
**Step 1**. Treatment with 0.3% Triton X-100 (in 0.01 M PBS, pH 7.4), 60 min (on shaker) at RT	Wash, 5 min, 4 times	Wash, 5 min, 4 times
Wash (with PBS), 5 min	**Step 4.** Visualization with Goat anti-mouse IgG (1:200), 75 min at RT	**Step 6.** Visualization with Goat anti-rat IgG (1:200), together with Hoechst (1:50,000), 75 min at RT
**Step 2**. Blocking with 5% NGS and 1% BSA, 60 min at RT	Wash, 5 min, 3 times	Wash, 5 min, 3 times
Wash, 5 min	**Step. 5.** Incubation with the rabbit primary (GFAP, 1:2), 60 min at RT and O/N at 4*C*	
**Step. 3**. Incubation with the mouse primary (NeuN, 1:500), 60 min at RT and O/N at 4°C		

*Remarks:1. Steps 1 and 2 can be combined together.2. Secondaries (Steps 4 and 6) can be replaced by donkey anti-mouse and -rat IgG.*

A variety of vendors sell secondaries that have been purified by “pre-adsorption” with IgG obtained from the potentially cross-reacting species. In this process, the secondary of interest is elluted through on a column in which IgG from the non-desired species has been attached to the beads of the column, with the hope that any cross-species binding secondaries present will bind to the column and allow the elution of exclusively non-cross-species binding secondaries. In order to ascertain if mouse-rat cross-species secondary binding could be blocked by pre-adsorption of the secondary as proposed (Erickson et al., 1993), single or double staining was performed with rat anti-GFAP and/or mouse anti-NeuN and visualized with goat anti-rat and/or goat anti-mouse secondary that had been highly cross-adsorbed to normal mouse or rat serum (Jackson Immuno Research), respectively. Stained slices were mounted on pre-cleaned slide glass, cover-slipped with aqueous mounting medium, and kept in the dark. Imaging was performed with an Olympus Fluoview 1000 system (Olympus America, Center Valley, PA), following the same confocal settings as previously optimized (Xiong et al., 2012; Mao et al., 2016).

## Results

### Same-Species vs. Cross-Species Visualization for Single Staining

**FIGURE 1:**
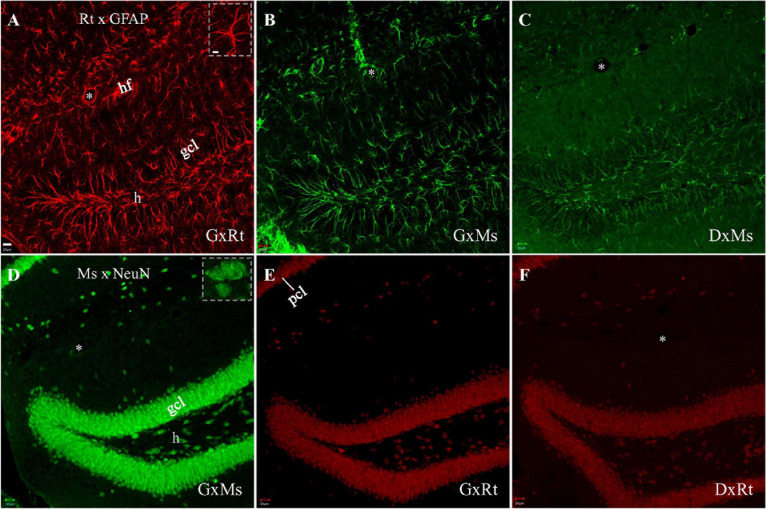
Single immunofluorescent staining with rat anti-GFAP or mouse anti-NeuN primary, visualized with same-species or cross-species secondaries. Each stack of confocal photomicrographs acquired in 10-μm-depth from a hippocampal slice with sequential scanning module. **(A–C)** Staining with rat anti-GFAP primary, visualized with goat anti-rat **(A)**, goat anti-mouse **(B)**, or donkey anti-mouse **(C)** secondary, respectively. Note the presence of numerous stained spiny astrocytic processes in **(A)**, and the absence of round, compact, neuron cell body-like staining. **(B,C)** Indicate unintended cross-species binding of anti-mouse secondary to rat primary, following the same staining pattern as in **(A)**. **(D–F)** Staining with mouse anti-NeuN primary, visualized with goat anti-mouse **(D)**, goat anti-rat **(E)**, or donkey anti-rat secondary **(F)**, respectively. Round compact neuronal nuclei and/or cell bodies are visible in **(D)**, and spiny astrocytic processes are absent. **(E,F)** Show unintended cross-species binding of anti-rat secondary to mouse primary. Higher magnification of specific GFAP-stained astrocytes or NeuN-stained neurons highlighted in (*Insets*) in **(A,D)**, respectively. DxMs/DxRt, donkey anti-mouse/donkey anti-rat secondary; gcl, granule cell layer; GxMs/GxRt, goat anti-mouse/goat anti-rat secondary; h, hilus; hf, hippocampal fissure; pcl, pyramidal cell layer. *Asterisks* indicating blood vessels in hf. Scale bars: 20 μm in all panels, except for 10 μm in (*Insets*).

We began our investigation of cross-species interaction between rat primary (anti-GFAP) and anti-mouse secondary, or mouse primary (anti-NeuN) and anti-rat secondary by performing single staining in order to establish a baseline for comparison to subsequent experiments combining both primaries. Rat anti-GFAP and mouse anti-NeuN primaries were employed because their antigens are exclusively present in different cell types, and therefore their distributions do not overlap. Fluorescent immunostaining with the rat anti-GFAP primary, visualized with its nominal same species secondary (i.e., goat or donkey anti-rat secondary), demonstrated numerous astrocytes with intensely stained fibrous branches (Figure 1A; *Inset*). Neurons were not stained by the rat anti-GFAP primary, supporting published reports (See et al., 2007; Smith et al., 2015). Astrocytic staining, however, could also be identified when rat anti-GFAP primary was visualized with the nominally non-rat binding goat anti-*mouse* secondary (Figure 1B). To determine if cross-species secondary binding could be produced by anti-mouse secondary raised from a source other than goat, we performed visualization for rat anti-GFAP primary using donkey-anti-mouse secondary. Figure 1C demonstrates that the donkey anti-mouse secondary also confirmed positive astrocytic staining, although this staining was not as strong as and the background was higher than visualization under goat anti-mouse secondary (Figure 1B).

To test if cross-species interaction could occur between the other primary-secondary cross-species pair (i.e., mouse primary and anti-rat secondary), we began as before by staining with a single primary, but this time using mouse anti-NeuN, and visualizing with the nominal same-species secondary (i.e., goat or donkey anti-mouse). This combination revealed bright neuronal staining without showing astrocytes (Figure 1D; *Inset*). When mouse anti-NeuN staining was visualized with goat anti-rat or donkey anti-rat secondary, cross-species neuronal staining could still be clearly seen (Figures 1E,F), although more dimly than with the same-species goat anti-mouse secondary (Figure 1D).

### Different Protocols for Double Staining With Rat and Mouse Primaries

**FIGURE 2:**
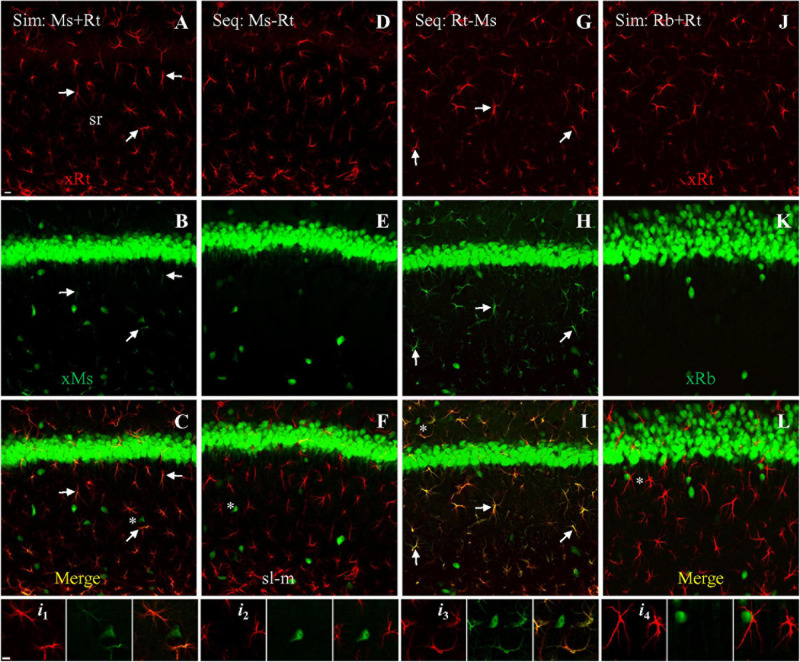
Double staining with both rat anti-GFAP and mouse anti-NeuN primaries using different protocols, visualized with goat-derived secondaries. Single confocal photomicrographs acquired from hippocampal area CA1. **(A–C)** Simultaneous staining (Sim) with a mixture of both primaries, visualized with a mixture of goat anti-rat (**A**, red) and anti-mouse (**B**, green) secondaries. Note in **(B)** that the anti-mouse secondary stained mouse anti-NeuN in neuron cell bodies as expected, but also stained the cross-species primary rat anti-GFAP in astrocytes. **(D–F)** First sequential protocol (Seq) by completing staining with mouse anti-NeuN primary (visualized with anti-mouse secondary), followed by staining with rat anti-GFAP primary (visualized with anti-rat secondary). Note the absence of astrocytic staining in E, indicating that mouse-then-rat sequential staining did not result in cross-species astrocytic staining of rat anti-GFAP by anti-mouse secondary. **(G–I)** Second sequential protocol by completing staining with rat anti-GFAP primary (visualized with anti-rat secondary), followed by staining with mouse anti-NeuN primary (visualized with anti-mouse secondary). Check the green channel **(B,E,H)** for cross astrocytic staining and merged channel **(C,F,I)** for co-staining as orange to yellow color (if any) from all these three protocols. *Arrows* indicating representative astrocytes co-stained by same- and cross-species binding. **(J–L)** As negative control for cross-species reaction, simultaneous staining with the rat anti-GFAP primary and a rabbit anti-NeuN primary, visualized with a mixture of goat anti-rabbit (green) and goat anti-rat (red) secondaries. No cross astrocytic staining could be seen **(K,L)**. Stained astrocytes and a nearby neuron from each staining protocol (asterisks) were highlighted in four groups **(*i*_1_–*i*_4_)** of three *Insets*, which indicate the red, green, and merged channel, respectively. Ms/Rb/Rt, (primary) raised in mouse, rabbit or rat; sl-m, stratum lacunosum-moleculare; sr, stratum radiatum; xMs/Rb/Rt, (goat) anti-mouse, -rabbit or -rat secondary. Scale bars: 10 μm in all panels.

In order to develop a method to block primary-secondary cross-species interaction when staining with both rat (GFAP) and mouse (NeuN) primaries on the same brain slices, we tested three different dual primary protocols: (1) simultaneous application of both primaries followed by simultaneous application of both secondaries, (2) sequential application of mouse primary then anti-mouse secondary, followed by rat secondary then anti-rat secondary, or (3) sequential application of rat primary then anti-rat secondary, followed by mouse primary then anti-mouse secondary. After simultaneous staining, astrocytes (Figure 2A, red) and neurons (Figure 2B, green) could both be clearly identified. However, simultaneous staining also resulted in cross-species astrocytic staining of the rat anti-GFAP primary by the anti-mouse secondary (Figure 2B, *Arrows*), visible in the merged panel as co-staining of astrocytes by both secondaries (Figure 2C, orange to yellow, *Arrows*). In summary, while both secondaries successfully targeted and bound to their intended species primary, the anti-mouse secondary also bound to the cross-species rat anti-GFAP primary (Figure 2*i*_1_).

We next tested sequential application of the two primaries. In the first sequential experiment we started staining for NeuN followed by GFAP staining (Table 2), we could see clear staining of astrocytes (Figure 2D, red) and neurons (Figure 2E, green), without any cross-species astrocytic staining in the green channel (by goat anti-mouse secondary to the rat anti-GFAP primary; Figure 2E). As such, no co-staining of astrocytes could be found (pure red or green only; Figures 2F*i*_2_). In the second sequential protocol we reversed the species order of the first sequential experiment. We found positive astrocytes (Figure 2G, red) and neurons (Figure 2H, green) as expected, but also obvious cross-species astrocytic staining (of rat anti-GFAP by the anti-mouse secondary; Figure 2H, green; *Arrows*). Therefore, co-staining of astrocytes by both secondaries was clearly visible in the merged image (Figure 2I, orange to yellow; *Arrows*). The later sequential protocol showed more intense cross-species staining of astrocytes (Figure 2*i*_3_) than the simultaneous protocol, likely due to the higher concentration of free anti-mouse secondary available for binding to rat anti-GFAP primary (compared to bound anti-rat secondary) in the visualization medium than was present for the simultaneous protocol. We did not observe cross-species *neuronal* staining (of mouse anti-NeuN by the goat anti-rat secondary) in any of the above three immunostaining protocols (Figures 2A,D,G*i*_1_–*i*_3_, red).

As a negative control for mouse-rat cross-species binding, we performed simultaneous staining with a mixture of a rabbit anti-NeuN and rat anti-GFAP primaries, visualized with a mixture of goat anti-rabbit and anti-rat secondaries. Unlike the mouse anti-NeuN primary, the rabbit anti-NeuN primary is more specific for neuronal nuclei and cell bodies because it is not cross-reacting with synapsin I in brain tissue (a marker for presynaptic boutons), as we showed previously (Mao et al., 2016). Intensely stained astrocytes (Figure 2J, red) and neurons (Figure 2K, green) could be seen, without cross-species staining of astrocytes or neurons (Figure 2*i*_4_). No co-staining of astrocytes could be identified in merged image (Figures 2L, *i*_4_).

### Single Staining Visualized With Dual Secondaries to Assess Competitive Binding

**FIGURE 3:**
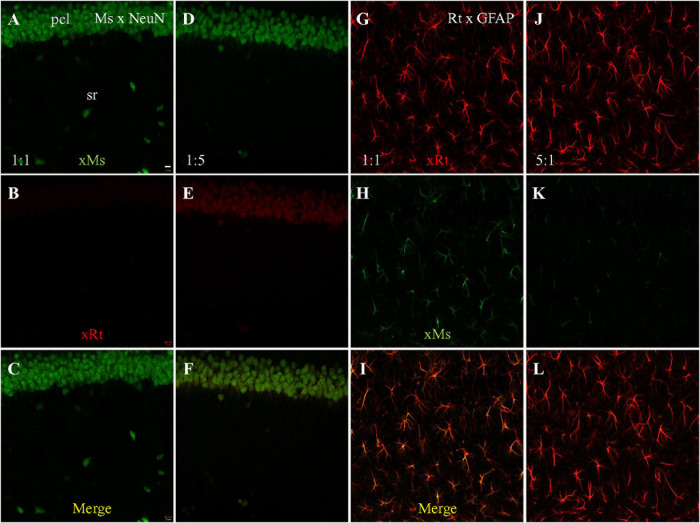
Single staining with mouse anti-NeuN or rat anti-GFAP primary, visualized with dual secondaries at different dilution. Single confocal photomicrographs acquired from hippocampal area CA1. **(A–C)** NeuN staining visualized with goat anti-mouse (green) and goat anti-rat (red) secondaries, both at dilution of 1:200. Strong same species neuronal staining is present in A as expected, and no cross-species staining is visible in **(B)**, suggesting that the same-species anti-mouse secondary out-competed the cross-species anti-rat secondary. **(D–F)** NeuN staining visualized with 1:500 of anti-mouse and 1:100 of anti-rat secondaries. Even at a lower concentration (1:500) neuronal same-species anti-mouse staining is still clearly visible in **(D)**. In panel **(E)**, even though no rat primary antibody was used, anti-rat secondary still shows faint staining, but in a cross-species neuronal pattern. **(G–I)** GFAP staining visualized with both secondaries at 1:200. **(J–L)** GFAP staining visualized with 1:100 of anti-rat and 1:500 of anti-mouse secondaries. Comparative dilution of same-species and cross species secondaries was shown in each immunostaining setting. xMs/xRt, (goat) anti-mouse, or anti-rat secondary. Scale bar: 10 μm for all panels.

To more directly investigate the possibility of competitive binding by the anti-mouse and anti-rat secondaries, we performed single staining followed by visualizing with both secondaries. Mouse anti-NeuN staining visualized with both secondaries at a 1:1 dilution ratio (both present at 1:200) showed specific neuronal staining by the anti-mouse secondary (Figure 3A green). We did not see cross-species neuronal staining by the anti-rat secondary (Figure 3B) or overlapping co-staining in the merged image (Figure 3C). Because our previous results suggested that the anti-mouse and anti-rat secondaries might have partially overlapping epitopes, and because the anti-mouse secondary had stronger cross-species staining than the anti-rat, we therefore tested a fivefold lower dilution (1:100; i.e., higher concentration) of anti-rat secondary compared to anti-mouse (1:500 dilution). This visualization resulted in both same-species (by the anti-mouse secondary; Figure 3D, green) and cross-species (by the anti-rat secondary; Figure 3E, red) neuronal staining. In summary, when the concentration of anti-mouse secondary was decreased, and the concentration of anti-rat secondary was increased, cross-species neuronal staining of mouse anti-NeuN primary by the anti-rat secondary was clearly visible (Figures 3E,F), suggesting competitive binding by the two secondaries to the mouse anti-NeuN primary.

Since our initial experiments had also demonstrated anti-mouse secondary cross-species binding to rat anti-GFAP primary, we also performed single staining with rat anti-GFAP primary, visualized with both anti-rat and anti-mouse secondaries. GFAP staining visualized with both secondaries at a 1:1 dilution ratio (1:200 for each) showed clear astrocytic same-species staining (by anti-rat secondary; Figure 3G, red) but also cross-species astrocytic staining (by anti-mouse secondary; Figure 3H, green). Furthermore, the merged image also clearly shows co-staining of astrocytes by both secondaries (Figure 3I, yellow). When the comparative dilution of anti-rat secondary was decreased to one fifth of the anti-mouse (Figures 3J–L), intense same-species astrocytic staining was present as expected (Figure 3J), but faint cross-species astrocytic staining by the anti-mouse secondary (Figure 3K, green) was still visible, although less strongly than in the 1:1 staining (Figure 3H). Due to the strong same-species staining of astrocytes by the anti-rat secondary (Figure 3J), co-staining of astrocytes by both secondaries could not be clearly seen in the merged image (Figure 3L). Taken together, the dual secondary visualization of single staining with rat anti-GFAP primary results showed less cross-species astrocytic staining by anti-mouse secondary when the concentration of anti-rat secondary was raised, suggesting that both secondaries competitively bound to rat primary in addition to competitively binding to mouse primary.

### Visualization With Pre-adsorbed Secondaries

**FIGURE 4:**
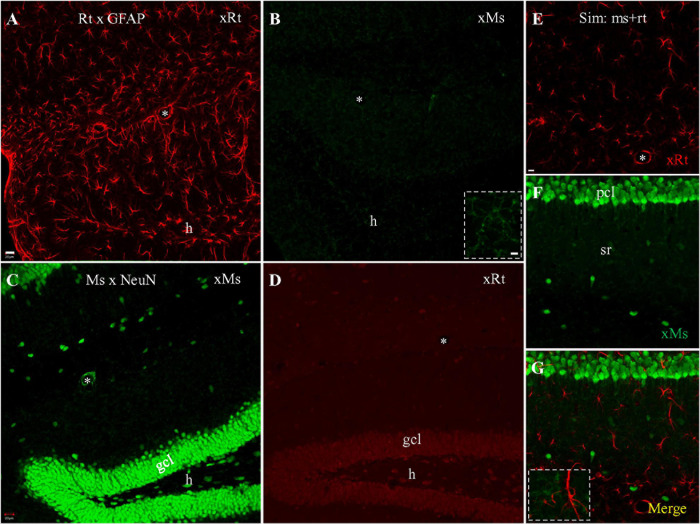
Single and double staining visualized with cross species pre-adsorbed secondaries raised from goat. **(A,B)** Stack of confocal photomicrographs acquired from the hippocampus stained with rat anti-GFAP primary and visualized with pre-adsorbed goat anti-rat **(A)** or anti-mouse **(B)** secondary. Note group of microglia faintly stained in **(B)** that were prominent under high magnification (*Inset*). **(C,D)** Stack of photomicrographs after staining with mouse anti-NeuN primary, visualized with cross species pre-adsorbed anti-mouse **(C)** or anti-rat **(D)** secondary. **(E–G)** Confocal photomicrograph showing simultaneous staining with rat anti-GFAP and mouse anti-NeuN primaries, visualized with a mixture of pre-adsorbed anti-rat (**E**, red) and anti-mouse (**F**, green) secondaries. Note the absence of co-staining of astrocytes or neurons. Distinct apposition of stained astrocytes (red) and microglia (green) could be clearly identified in merged image (**G**, *Inset*). *Asterisks* indicating blood vessels in hf. Scale bars: 20 μm in **(A–D)**; 10 μm in **(E–G)**; 5 μm in *Insets*.

An alternative solution to the problem of cross-species binding by secondary antibodies is to run the secondary antisera down a column in which cross-species primary antisera has been attached to the beads of the column, in the hope that any cross-species binding secondaries will bind to the column while species-specific secondaries elute through. To determine if cross-species adsorption of the secondary could block mouse-rat cross reaction as proposed (Erickson et al., 1993), we visualized staining with rat anti-GFAP and mouse anti-NeuN primaries separately, and combined, using these special pre-adsorbed secondaries. Single staining with rat anti-GFAP primary visualized with pre-adsorbed anti-rat secondary resulted in intensely and clearly stained astrocytes (Figure 4A), as expected. When visualized with pre-adsorbed anti-mouse secondary (Figure 4B) we could not see cross-species staining of astrocytes. Instead, large numbers of microglia, which do not contain GFAP, were stained throughout the sections for unknown reasons (Figure 4B), and could be clearly identified under high magnification (Figure 4B; *Inset*). Single staining with mouse anti-NeuN primary visualized with pre-adsorbed anti-mouse secondary produced intense same-species neuronal staining, and little if any staining of microglia (Figure 4C). Cross-species neuronal staining could still be seen when this staining was visualized with pre-adsorbed goat anti-rat secondary (Figure 4D), although in this case cross neuronal staining was much weaker than the visualization with regular goat anti-rat secondary (Figure 1E). When simultaneous staining with rat anti-GFAP together with mouse anti-NeuN primaries was visualized with both pre-adsorbed secondaries (Figures 4E–G), we encountered clear and separate staining of astrocytes (Figure 4E, red) and neurons (Figure 4F, green). No cross-species astrocytic staining of rat anti-GFAP (by pre-adsorbed anti-mouse secondary) or cross neuronal staining of mouse anti-NeuN (by pre-adsorbed anti-rat secondary) was evident (Figures 4E–G). Instead, distinct apposition of intensely stained astrocytes (red) and faintly stained microglia (green) could be clearly identified (Figure 4G and *Inset*), in addition to brightly stained neurons nearby (Figures 4F,G, green). In summary, pre-adsorption of anti-mouse secondary could block cross-species visualization of astrocytes by rat anti-GFAP primary (but showed unexplained binding to microglia). However, the cross-species staining of neurons by anti-rat secondary binding to mouse anti-NeuN primary was just partially blocked, suggesting that its pre-adsorption might be incomplete.

## Discussion

We developed a sequential staining protocol that alleviates the problem of cross-species binding by anti-mouse and anti-rat secondaries during dual primary antibody staining experiments (i.e., anti-mouse secondary binding to rat primary, and anti-rat secondary binding to mouse primary are blocked). We predict that our protocol will find widespread use when species-specific secondaries either do not exist or are not readily available. In particular, we found cross-species reactivity between a rat primary (anti-GFAP) and a goat anti-mouse secondary similar to previous reports (Erickson et al., 1993). Furthermore, we showed similar cross-species binding when replacing the goat-derived anti-mouse secondary with a donkey-derived anti-mouse, suggesting that the animal species producing the anti-mouse secondary was not a determinant factor of this cross-species binding. We also observed cross-species binding when staining with a mouse primary (anti-NeuN) followed by visualizing with goat or donkey anti-rat secondary, indicating that both primaries could be recognized by both secondaries. Further study will be needed to determine the basis and magnitude of both cross-species interactions.

To develop a double staining technique that blocks cross-species interaction, we compared three different protocols using mouse anti-NeuN and rat anti-GFAP primaries: simultaneous application of both primaries followed by simultaneous visualization with both secondaries; mouse primary and anti-mouse secondary followed by rat primary and anti-rat secondary (Table 2); and rat primary and anti-rat secondary, followed by mouse primary and anti-mouse secondary. To increase the rigor of our findings and highlight the usefulness of our method, we chose secondaries raised from goat because they appeared to show stronger cross-species binding to the primaries than did donkey-produced secondaries (Figure 1). With simultaneous staining, strong cross-species binding was observed from anti-mouse secondary to the rat primary (anti-GFAP). Sequential staining starting from rat anti-GFAP primary and anti-rat secondary, followed by mouse anti-NeuN primary and anti-mouse secondary, resulted in cross-species staining of astrocytes (but not neurons) by the nominal anti-mouse secondary binding to the rat primary, similar to the cross-species astrocytic staining of the simultaneous protocol. Importantly, and the major finding of this work, is that no cross-species staining of either astrocytes or neurons could be found when sequential staining started from mouse anti- NeuN primary and anti-mouse secondary, followed by rat anti-GFAP primary and anti-rat secondary (Table 2). That is, in the dual primary experiments, if anti-*mouse* secondary was present at a working concentration when *rat* primary was also present, then cross-species binding of *rat* primary by anti-*mouse* secondary was seen. These findings lead us to speculate that a competition exists between the anti-mouse and anti-rat secondaries in their binding to both primaries, with binding of anti-mouse secondary to mouse primary being the strongest and anti-rat secondary to mouse primary the weakest (Table 3). Because the anti-rat was the weaker secondary, we tested single staining with mouse anti-NeuN primary or rat anti-GFAP visualized with dual secondaries for competition by raising the concentration of anti-rat secondary and lowering the concentration of anti-mouse secondary. Doing so resulted in the appearance of cross-species neuronal binding by the nominal anti-rat secondary to the mouse anti-NeuN primary (in the presence of anti-mouse secondary). Raising the relative concentration of anti-rat secondary also reduced the cross-species binding of anti-mouse secondary to rat anti-GFAP primary (Figure 3), further suggesting that the secondary antibody competition may be dosage-dependent.

**TABLE 3:** Hypothetical scaling of anti-mouse or anti-rat secondaries in binding ability to mouse and rat primaries.

	**Anti-mouse secondary**	**Anti-rat secondary**
Mouse primary	+ + +	+
Rat primary	+ +	+ +

*Remarks: + indicating binding ability. The more +, the stronger binding between the primary-secondary pair.*

**FIGURE 5:**
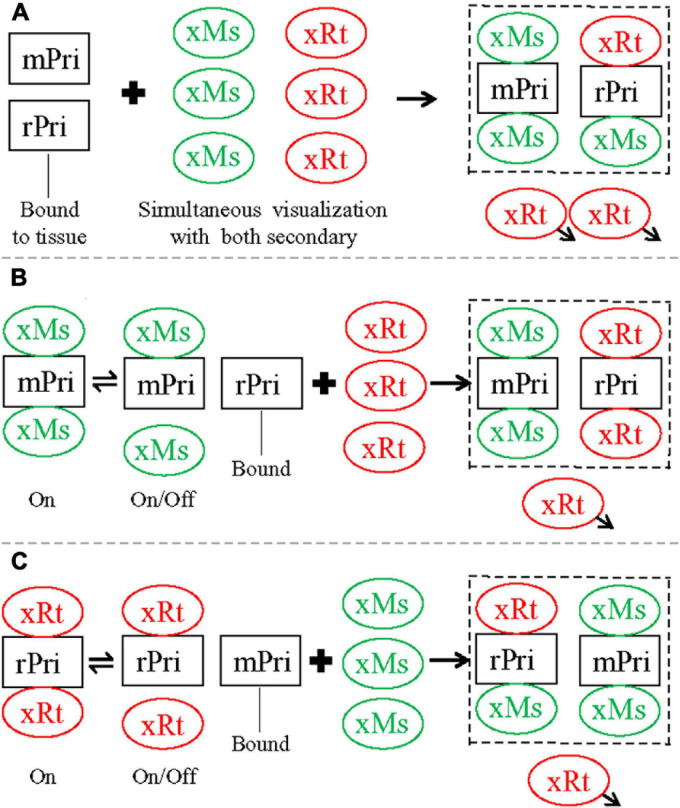
Summary diagram illustrating staining pattern of different double staining protocols tested in the present study, closely related to Figure 2. **(A)** Simultaneous protocol. After incubation, both mouse (mPri) and rat (rPri) primaries were bound to their designed antigens in tissue slices, and the excessive primaries washed away. Simultaneous visualization was then performed with a mixture of anti-mouse (xMs) and anti-rat (xRt) secondary. xMs would bind tightly to mPri for same-species reaction as expected. The excessive xMs would also compete well enough at binding to rPri to produce cross-species staining. xRt, by contrast, bound well to rPri for same-species staining but could not compete well against xMs for cross-species binding to mPri. As a result, mPri could be seen as pure green by xMs only, whereas rPri appeared as co-staining with both red (from xRt) and green (from xMs) fluorescence. When the staining stopped, the excessive secondary would be washed away (*arrowheads*). **(B)** Sequential protocol 1. Slices were first incubated with mPri and visualized with xMs consecutively, with excessive antibodies of this pair washed away. After second round incubation with rPri, xRt was used for visualization. Although xMs that was already bound to mPri would shift between on and off status, the excessive xRt in visualization medium would not displace xMs for cross-species binding to mPri, since xRt has weaker binding ability to the mouse primary (as speculated above). As a result, mPri kept being stained as pure green from xMs. rPri, on the other hand, would only be stained as red from xRt since there was no extra xMs this time for cross-species binding to rPri. **(C)** Sequential protocol 2. Slices were first stained with rPri and visualized with xRt consecutively, with excessive antibodies of this pair washed away. After second round of incubation with mPri, xMs was used for visualization. xRt that was already bund to rPri would shift between on and off status at this time. While xMs preferred to bind to mPri, the extra xMs in visualization medium would displace some of xRt for cross-species binding to rPri. As a result, rPri that had been coupled with xRt (red) would be changed to co-staining as red and green from both secondary and mPri as pure green again from xMs. The displaced xRt then would be washed away when the whole staining process was stopped. *Dashed squares* indicate the final staining pattern by each protocol.

Therefore, we propose the following mechanism to explain our cross-species staining, and the sequential staining protocol we developed that blocks this cross-species staining. When single staining was performed with either mouse anti-NeuN or rat anti-GFAP primary, it could be recognized by either secondary used alone (either anti-mouse or anti-rat, as in Figure 1), i.e., both secondaries were capable of binding both primaries. Antibody-antigen binding is the net result of a dynamic equilibrium between on (association) and off (dissociation; Reverberi and Reverberi, 2007), and at any given moment some fraction of the primary will be bound by secondary in visualization medium, and some will not. As this is an equilibrium reaction, you can increase (or decrease) the fraction of secondary bound to primary by increasing (or decreasing) the concentration of secondary, and a typical protocol strikes a balance between raising the secondary concentration as much as possible to push this equilibrium, while keeping it as low as possible to avoid non-specific binding. A tightly binding antibody, present to excess and without strong competition, would be expected to bind a very high fraction of its antigen (e.g., anti-mouse secondary used alone against mouse primary). When two antibodies (in this case secondaries) compete for the same antigen (the primary), equilibrium will develop in which the relative fraction of antigen bound by the different antibodies depends on the relative affinities of the antibodies for their antigens, and the relative concentrations of the antibodies. In the case of simultaneous staining (for mouse and rat primaries together, visualized by anti-mouse and anti-rat secondaries together), anti-mouse secondary binds tightly to mouse anti-NeuN primary as expected, but also competes well enough at binding to rat anti-GFAP primary to produce cross species astrocytic staining. Anti-rat secondary, by contrast, binds well to rat anti-GFAP primary for same-species astrocytic staining but does not compete well against anti-mouse secondary at binding for mouse anti-NeuN primary for neuronal staining. As a result, mouse anti-NeuN staining for neurons could be seen as pure green by anti-mouse secondary only, while rat anti-GFAP for astrocytes appeared as co-staining with both red (anti-rat secondary) and green (anti-mouse secondary) fluorescence, as summarized in Figure 5A. Sequential staining exploits competition between two secondaries in binding to both primaries, by ensuring that high concentrations of the cross-species binding anti-mouse secondary (the stronger secondary) are not present at the same time as rat primary. When the mouse primary against NeuN is stained and visualized by anti-mouse secondary, the mouse secondary is rinsed extensively and thoroughly before the rat primary is applied. In our double primary experiments, during the successful sequential staining protocol (mouse primary and anti-mouse secondary, followed by rat primary and anti-rat secondary), we propose that the anti-rat secondary did not bind strongly enough to the mouse primary to dissociate an observable amount of anti-mouse secondary from the mouse primary, and therefore staining for the mouse and rat primaries did not overlap (Figure 5B). By contrast, in the unsuccessful sequential staining protocol (rat primary and anti-rat secondary, followed by mouse primary and anti-mouse secondary), the later added anti-mouse secondary binds tightly to free mouse primary (anti-NeuN) as expected. However, this anti-mouse secondary also binds tightly enough to rat primary (anti-GFAP) to displace a noticeable amount of dissociated anti-rat secondary. As such, some rat anti-GFAP staining in astrocytes

could be visualized by both anti-rat (red) and anti-mouse (green) secondaries (Figure 5C). We encountered more intense cross-species staining of astrocytes after this unsuccessful sequential protocol than after the simultaneous protocol (Figures 2A–C,G–I), supporting the above speculation that anti-mouse and anti-rat secondaries may compete with each other for binding to primaries in a dosage-dependent manner. During the second visualization (nominally for anti-mouse secondary binding to mouse anti-NeuN primary) when anti-rat secondary already bound to rat anti-GFAP primary and excessive free anti-rat secondary was washed away, there should be more anti-mouse secondary available for cross-species binding than during the dual visualization in simultaneous protocol. Cross-species neuronal staining (by anti-rat secondary binding to mouse anti-NeuN primary) was never observed in any of the three double primary protocols (Figure 2), consistent with our speculation that the binding ability of anti-rat secondary (red) for mouse anti-NeuN primary is much weaker than that of anti-mouse secondary (green) for the same mouse primary.

It has been proposed that cross-species binding by secondaries can be blocked by using pre-adsorbed secondaries that have been purified by initially eluting them through a column in which serum IgG from the cross-reacting species has been attached to the beads of the column. In theory, any secondary with an affinity for the cross-reacting species will thus bind to the column, while non-cross-reacting secondaries will pass through. For instance, anti-mouse secondary (anti-mouse IgG) could be purified by pre-adsorption against normal rat IgG to prevent the secondary from cross-species binding to rat primaries (IgG; Erickson et al., 1993). We verified here that pre-adsorption of the anti-mouse secondary (with rat serum, according to data sheet from Jackson Immuno Research) could block cross-species astrocytic staining (of rat anti-GFAP primary) by the pre-adsorbed anti-mouse secondary. However, cross-species neuronal staining (of mouse anti-NeuN primary) by the pre-adsorbed anti-rat secondary could not be completely blocked by pre-adsorption of this secondary (with mouse serum), suggesting that its pre-adsorption might be incomplete. We demonstrated here that the strength of cross-species binding between anti-rat secondary to mouse anti-NeuN primary might be much lower than that of anti-mouse secondary to rat anti-GFAP primary. When using the same protocol for a complete pre-adsorption for the anti-mouse secondary, the anti-rat secondary might only be partially pre-adsorbed. However, we do not know yet if improvement in the pre-adsorption of the anti-rat secondary can be reached by extending processing time and/or by increasing the concentration of mouse IgG to the beads. Nonetheless, in double staining experiments with the pre-adsorbed secondaries, cross-species astrocytic staining was not detected, as shown in simultaneous staining and visualizing with regular secondaries together (Figures 2A–C). Incomplete pre-adsorption of the goat anti-rat secondary would not result in incorrect double staining results, since the pre-adsorbed anti-mouse secondary here is the key factor for the cross reaction when performing double immunostaining.

In contrast to the original report on cross-species binding of anti-mouse secondary to rat primaries (mouse-rat cross reaction; Erickson et al., 1993), we found the reverse cross-species binding problem, i.e., anti-rat secondary binding to mouse primary. This new finding has not been reported previously, likely due to the fact that the rat-mouse cross reaction was concealed behind the mouse-rat cross reaction during double staining, as we showed using simultaneous staining protocols. We also demonstrated a complete pre-adsorption of goat anti-mouse secondary by normal rat serum and a partial pre-adsorption of goat anti-rat secondary by normal mouse serum. However, the incomplete pre-adsorption of the anti-rat secondary did not interfere with our results from the simultaneous staining using both mouse and rat primaries under visualization by dual pre-adsorbed secondaries. This observation provides additional evidence for our hypothesis that the anti-rat secondary is not the determining factor in the cross-species reaction during double staining with both mouse and rat primaries. Our sequential staining protocol (as shown in Table 2) can be adopted when pre-adsorbed secondaries are not readily available. Furthermore, the complicated pre-adsorption process of secondaries may not be necessary when dealing with mouse-rat cross-species binding, since our successful sequential protocol yields staining results at least as good as the pre-adsorption protocol.

When indirect double staining is necessary, it is best to use primaries from different animal species for which the secondaries do not typically cross-react (e.g., mouse and rabbit, but not mouse and rat), and this can be verified by staining with each primary alone under visualization with the secondary not directed against that primary. Unfortunately, primaries meeting the above requirements might not exist, or might not be readily available. For example, among the nine available primaries against amyloid precursor protein, only one is neuron specific (Guo et al., 2012). In situations where the availability of primaries is limited, double staining with a pair of working primaries produced in cross-reacting species (e.g., mouse and rat) may be inevitable. When this occurs, sequential staining may work, starting from the primary with strong cross-species secondary binding that may be suppressed by its strongest same-species binding, followed by staining with the primary whose cross-species secondary binding was weak (as listed in Table 2 for the primary and secondary pairs we tested here). For visualization of the second primary (rat anti-GFAP), using the anti-rat secondary at a slightly lower concentration (or higher dilution such as 1:500) might be helpful in order to avoid its potential binding to the first primary (mouse anti-NeuN). The concentration of the first (anti-mouse) secondary has been greatly decreased at this point, due to the thorough wash at the end of first round of staining-visualization (see Materials & Methods). This might comparatively raise the concentration of the later (anti-rat) secondary and possibly induce the reverse cross-reaction by anti-rat secondary to mouse anti-NeuN primary, similar to Figure 3E. However, the concentration of this secondary should not be set too low (for example, 1:1,000). Otherwise, correct staining might not be guaranteed when “thick” slices are used, as in the present study. We also recommend searching for a secondary antibody host with the least cross-species secondary binding (in our study this would have been donkey anti-mouse and anti-rat secondaries). The technique we established here to block mouse-rat cross species staining will be of widespread use when compatible primary antibodies do not exist, or are not readily available. Further study may be needed to determine if this technique works for other cross-species binding pairs as well (e.g., guinea pig and rabbit).

The mouse-rat and rat-mouse cross-reaction highlighted in the present study may provide warning of an additional problem when *single* immunostaining is performed in special tissues where high levels of endogenous primaries may be present. Examples would include slices from the rat immune system or in some pathological conditions including inflammation or bleeding. In such cases, interfering staining might be acquired if a mouse primary is used for staining and an anti-mouse secondary (with some affinity for rat primaries) is used for visualization. The desired target might actually be missing completely, and a wrong interpretation might be reached. When a rat primary is used to stain similar slices from the mouse, we have to be especially cautious to the reverse cross-reaction we found, i.e., cross-species binding from an anti-rat secondary to mouse primaries. To reveal if mouse-rat or rat-mouse cross-reaction occurs, we can incubate these slices solely with the secondary to be used. When positive staining is still manifested, a shift of the primary to the one produced from other animal species would be an ideal troubleshooting option.

## Data Availability Statement

The raw data supporting the conclusions of this article will be made available by the authors, without undue reservation.

## Ethics Statement

The animal study was reviewed and approved by Institutional Animal Care and Use Committees of Wuhan University, Children’s Hospital of Philadelphia and University of Pennsylvania.

## Author Contributions

SM and GX performed the experiments, collected, and analyzed. SM, GX, BJ, and NC interpreted the data. SM and AC revised the manuscript. GX and AC designed the study. GX wrote the manuscript. BJ and NC revised the manuscript. All authors approved the final version and agreed to be accountable for all aspects of the work.

## Conflict of Interest

The authors declare that the research was conducted in the absence of any commercial or financial relationships that could be construed as a potential conflict of interest.
